# Resuscitation room blood alcohol concentrations: one-year cohort study

**DOI:** 10.1136/emj.2008.062711

**Published:** 2008-10-16

**Authors:** R Touquet, E Csipke, P Holloway, A Brown, T Patel, A J Seddon, P Gulati, H Moore, N Batrick, M J Crawford

**Affiliations:** 1 Imperial College Healthcare NHS Trust, London, UK; 2 Central and North West London Foundation Trust, London, UK; 3 Imperial College London, London, UK

## Abstract

**Objective:**

To clarify the relationship between presenting clinical condition and blood alcohol concentration (BAC) among adult patients admitted to a resuscitation room (RR) of an emergency department (ED) in order to help guide clinical practice.

**Method:**

Single-site prospective cohort study of all patients admitted to the RR of an inner-city hospital over a one-year period. The study sample comprised all those aged 16 years and over from whom a blood sample was taken, with BAC (results not known to ED staff), pathology by International Classification of Diseases (ICD) version 10 coding, injury severity score for trauma, return visit to hospital and mortality during the subsequent 6-month period, being recorded.

**Results:**

291 (15%) of 1908 presentations had a positive BAC (ie, BAC >10 mg/100 ml) ranging from 11 to 574 mg/100 ml, of which almost 40% were over 240 mg/100 ml (ICD-10 code Y90.8). In addition to collapse from alcohol/drugs, almost half of those presenting following self-harm or assault had a positive BAC. Those with a positive BAC had a higher rate of ED re-attendance in the following 6 months. 10% of all presentations were due to trauma.

**Conclusion:**

The following five presentations to the RR are associated with a positive BAC: collapse from alcohol/drugs, self-harm, trauma, gastrointestinal bleeding (ICD-10 code K92.2) and non-cardiac chest pain (ICD-10 code R07). Patients with a positive BAC demonstrate a very wide range of pathology, some with severe levels of misuse. This highlights the opportunity for prompt feedback when sober, to ensure all is done to encourage patients to contemplate change in order to reduce re-attendance.

This work follows directly on from our first paper: “Use of blood alcohol concentrations in resuscitation room patients”,^1^ which covers ethical, judicial and insurance issues. Alcohol misuse is a very common problem confronting all healthcare staff working in emergency departments (ED) worldwide.^2^ The value of screening and brief intervention in ED by an alcohol nurse specialist is established,^3^ with one less return visit to the ED over 12 months for every two people referred to an alcohol nurse specialist.^4^ At our hospital early identification of alcohol misuse is by the Paddington alcohol test (PAT) facilitating brief advice (with possible referral for brief intervention).^5^

Identification of patients with alcohol problems in the ED may be by history (PAT), clinical examination or blood alcohol concentration (BAC). PAT application is usually not possible for patients who are potentially critically ill and the need is for urgent assessment and treatment. Clinical examination for possible alcohol use is limited for the obtunded patient and has variable concordance with BAC.^6^ A degree of tolerance, thereby camouflaging clinical signs, for the “experienced” drinker is also well recognised.^7^ Measurement of BAC may provide a means of assessing the influence of alcohol use on the person’s presentation and management,^8^^–^10 but our ED staff were blinded to results (BAC requests not previously being routinely available at our hospital). Requesting the BAC without prior consent is acceptable to potentially critically ill patients, provided that feedback is given when patients are in a sufficiently improved clinical state.^1^ ^11^

The extent of concurrent alcohol use among all categories of patients treated specifically in the resuscitation room (RR) of an ED has not previously been reported; every ED in the UK has a specific RR, usually between two and six bays, for potentially critically ill patients.^12^ We therefore set out to determine which RR patients were most likely to have a positive BAC and to explore associations with clinical outcomes, now that early identification with the giving of brief advice has been shown to be effective in reducing ED re-attendance.

## METHODS

The study took place in the four-bayed adult RR of the ED of an acute hospital serving a metropolitan inner-city population of over 500 000 adults, seeing 65 000 new adult (16 years and over) ED visits each year. We measured BAC over a 12-month period from all blood samples taken from patients initially presenting to the RR, with the exception of direct admissions to specialists and transfers from other hospitals (before this BAC testing was not available on our hospital site). The spectrum of disease of those patients presenting to the RR was broad (see tables 1 and 2). All had life-threatening, or potentially life-threatening pathology, triage categories 1 or 2.^12^ Patients under 16 years of age were excluded. The medical and nursing staff were blinded to BAC results, which therefore had no influence on patient management. The local research ethics committee approved this study as an audit, therefore informed consent was waived (no EC2143 dated 31 March 2005).

**Table 1:** Characteristics of 2137 episodes in which BAC was and was not tested

	Not tested	Tested	Total
	N = 229	N = 1908	N = 2137
Gender (male)*	106 (46%)	739 (39%)	845 (40%)
N = 2137			
Mean age (SD)	56.80 (20.52)	55.64 (21.04)	56.67 (20.58)
N = 2137			
Ethnicity (British white)	98 (43%)	803 (42%)	901 (42%)
N = 1912			
Computed tomography scan (n % yes)	30 (13%)	311 (16%)	341 (16%)
N = 2112			
Intubation (n % yes)	8 (4%)	76 (4%)	84 (4%)
N = 2115			
Median GCS score (range)	15 (12)	15 (14)	15 (14)
N = 2081			
Diagnosis (ICD code) N = 2137			
External causes of morbidity (V01–Y98)	39 (17%)	235 (12%)	274 (13%)
Diseases of the circulatory system (I00–I99)	84 (37%)	776 (41%)	860 (40%)
Diseases of the respiratory system (J00-J99)	25 (11%)	287 (15%)	312 (15%)
Diseases of the nervous system (G00–G99)	15 (6%)	101 (5%)	116 (5%)
Mental and behavioural disorders (F00–F99)	11 (5%)	90 (5%)	101 (5%)
Diseases of the digestive system (K00–K93)	10 (4%)	115 (6%)	125 (6%)
Other	45 (20%)	304 (16%)	349 (16%)

*p<0.01. BAC, blood alcohol concentration; GCS, Glasgow coma scale; ICD, International Classification of Diseases.

**Table 2:** Characteristics of 1908 episodes with a positive and negative BAC

	Alcohol <10 mg/100 ml	Alcohol >10 mg/100 ml
	N = 1617 (85%)	N = 291 (15%)
Gender (male)*	948 (59%)	251 (86%)
Mean age (SD)*	58.85 (20.47)	45.38 (17.63)
Ethnicity (British white)	654 (40%)	149 (51%)
Reason (ICD code)	N and % for each code	
External causes of morbidity (V01–Y98)		
Road traffic accident (V01–99)	43 (81%)	10 (19%)
Other accidents (W00–X59)	53 (62%)	36 (38%)
Assault (X85–Y09)	28 (57%)	21 (43%)
Self-harm (X60–X84)	23 (51%)	22 (49%)
Diseases of the circulatory system (I00–I99)		
Ischaemic heart disease (I20–I25)	358 (90%)	40 (10%)
Arrhythmic/conduct disorders (I44–I45)	110 (95%)	6 (5%)
Congestive cardiac failure (I50)	69 (93%)	5 (7%)
Myocardial infarction (I21)	46 (94%)	3 (6%)
Cerebral infarct/haemorrhage (I60–I64)	67 (92%)	6 (8%)
Other	60 (91%)	6 (9%)
Diseases of the respiratory system (J00–J99)		
Chronic obstructive pulmonary disease (J43–J44)	70 (96%)	3 (4%)
Asthma (J45)	51 (90%)	6 (10%)
Bronchitis/emphysema (J40–J42)	64 (97%)	2 (3%)
Other	85 (94%)	6 (6%)
Diseases of the nervous system (G00–G99)		
Paroxysmal disorders (epilepsy) (G40–G47)	74 (88%)	10 (12%)
Other	16 (94%)	1 (6%)
Mental and behavioural disorders (F00–F99)		
Alcohol misuse (F10)	4 (9%)	41 (91%)
Drug misuse (F11–F19)	13 (59%)	9 (41%)
Acute confusional state (F04)	5 (100%)	0 (0%)
Other	14 (78%)	4 (22%)
Diseases of the digestive system (K00–K93)		
Gastrointestinal bleed (K92.2)	34 (77%)	10 (23%)
Other	65 (92%)	6 (8%)
Other		
Acute infection/sepsis (A00–B99)	35 (88%)	5 (12%)
Non-cardiac chest pain (R07)	45 (76%)	14 (24%)
Syncope (R53)	32 (97%)	1 (3%)
Obstetric/gynaecological (O00–O99)	9 (90%)	1 (10%)
Anaphylaxis (T78.2)	20 (83%)	4 (17%)
Miscellaneous	124 (90%)	13 (10%)

*p<0.01. BAC, blood alcohol concentration; ICD, International Classification of Diseases.

Blood samples were collected into a fluoride-oxalate bottle (as for blood sugar estimation); BAC was measured by an automated enzymatic method—DRI ethyl alcohol assay (Microgenics GmbH, Passau, Germany) on an Olympus AU2700 analyser (Olympus Optical Co Ltd). BAC are reported in mg/100 ml; less or equal to 10 approximated to BAC negative and more than 10 was BAC positive.

Electronic and written ED and hospital records were subsequently examined by researchers masked to the BAC results. For those patients transferred to seven other hospitals, records were similarly examined for length of stay and outcome. In addition to basic demographic information, the time, date and reason for presentation to the RR were recorded, together with Glasgow coma scale (GCS) scores, information about immediate management (intubation/whether they received a computed tomography scan), final primary diagnosis (using International Classification of Diseases (ICD) version 10 codes^13^ for consistent standardisation), length of stay, return visit(s) to the ED and mortality over the following 6 months.

Trauma severity was rated by an independent team from the UK trauma audit and research network, commissioned for this study. All trauma cases were assessed using the injury severity score. This method uses the abbreviated injury scale scores based on the severity of the injury, which is applied to the regions of the body.^14^ The minimum score is 0, whereas the maximum score is 75. An injury severity scale score of less than 12 was used to classify minor trauma.

Each blood sample was assigned an identifying number to log the BAC results into an Excel spreadsheet that was not accessible by the treatment team. All data were then entered onto a database and analysed using SPSS (version 14.0). Univariate and multivariate tests were used to compare the characteristics and outcomes of those with positive and negative BAC. The impact of a positive BAC on management and outcomes was examined using binary logistic regression. For rates and proportions, 95% CI were calculated.

## RESULTS

**Figure 1:**
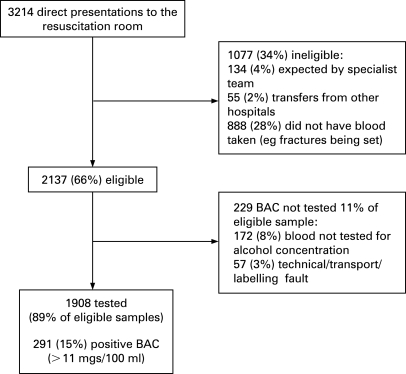
Flow diagram. BAC, blood alcohol concentration.

There were 2326 direct patient presentations to the RR during the course of the study, 22 August 2005 to 31 August 2006. Of these, 2137 patients were eligible to participate, but 229 of these were not tested (fig 1). The characteristics of those who were tested versus those who were not tested are presented in table 1. There were no differences between the groups except that men were less likely to have BAC tested.

Of the 1908 with BAC results, 1617 had BAC scores of less than 11 mg/100 ml (BAC negative). The remaining 291 (15%) had a positive BAC, ranging from 11 to 574 mg/100 ml. The characteristics of those BAC negative are compared with those with a positive test in table 2. The top five RR presentations associated with a positive BAC were collapse due to alcohol/drugs, self-harm, trauma, gastrointestinal bleeding and non-cardiac chest pain. An independent samples t test showed that those who were BAC positive had a mean age of 45.4 years compared with 58.9 years among those who were BAC negative (difference in means 4.99; 95% CI 10.97 to 15.96). Using binary logistic regression, men were found to be more likely to be positive than women (odds ratio (OR) 2.27, 95% CI 1.17 to 3.03).

**Figure 2:**
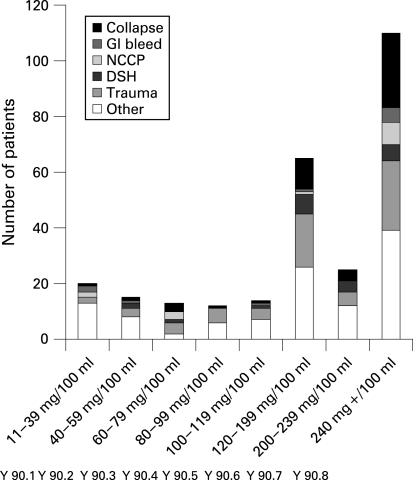
Blood alcohol concentration: prevalence by Y90 coding. DSH, deliberate self-harm; GI, gastrointestinal; NCCP, non-cardiac chest pain.

BAC were divided into ICD-10 diagnoses (table 2) and Y90.1–8 (BAC) codes (fig 2). A total of 110 (38% of 291 positives) had a BAC of over 240 mg/100 ml (Y90.8), of which 62 were over 300 mg/100 ml (21%), 18 were over 400 mg/100 ml (6%) and four were over 500 mg/100 ml (1%).

In our study, collapse due to alcohol misuse was classified under mental and behavioural disorders, F10 (F10.0 acute intoxication: with 10.1 harmful use, 10.2 dependent use, 10.3 withdrawal state).^13^ Of the 45 such patients, four (9%) had a BAC of less than 11 mg/100 ml. Of the 22 who were classified as primarily drug abuse (F11–F19), nine (41%) had a positive BAC, the association between the two being well recognised. Alcohol misuse may also be coded either Y90 (fig 2), with subcodes defined by a series of nine BAC levels, or Y91 by grades of clinical assessment in the absence of BAC.

Using binary logistic regression, the 196 incidents involving trauma (accidents, assault or self-harm—comprising only 10% of 1908) were found to be more likely to be BAC positive than other presentations (OR 3.52; 95% CI 2.54 to 4.0), apart from collapse due to alcohol/drugs. Those with a high trauma score were no more likely to be BAC positive than those with a low score: the mean BAC in those with a score below 12 was 68.1 compared with 76.6 in those with a trauma score above 12 (difference in means 8.5; 95% CI 0.46 to 2.45). Nearly half (46%) of patients with head and neck trauma were BAC positive, which demonstrated a trend towards significance (OR 1.91; 95% CI 0.97 to 3.82).

Running a binary logistic regression, those BAC positive were found to be more likely to have been intubated while in the resuscitation room (OR 1.80; 95% CI 0.99 to 3.27), even after taking into account the confounding effect of their presenting condition. However, they were not more likely to have been sent for a computed tomography scan (OR 1.17; 95% CI 0.79 to 1.73). Of the 1858 (97.4%) who had a GCS score documented in their notes, the median score was lower when BAC positive (z  =  −5.26, p<0.001). A total of 1412 (74%) of the 1908 presentations to the RR resulted in admission to hospital.

Regarding 6-month outcomes, of the 1753 patients (155 were return visits) who presented to our RR and had a BAC measured, 494 (28%) subsequently visited our ED during the following 6 months. It is notable that 219 (12%) died within 6 months, according to computer data held by our hospital (which records later patient death for those previously attending our ED). When taking into account the confounding effects of age and presenting condition, a positive BAC was associated with an increased likelihood of a return visit to the ED within 6 months (OR 1.46; 95% CI 1.03 to 2.05). The likelihood of admission to a ward on first attendance to the RR (OR 1.12; 95% CI 0.80 to 1.58) or death within 6 months (OR 1.21; 95% CI 0.66 to 2.21) were also higher among those with a positive BAC, but these were not statistically significant.

## DISCUSSION

This is the first such report assessing BAC taken over one year specifically from RR patients. We show that more than one in seven people treated in our RR over a 12-month period have consumed alcohol before their presentation. Half of all those treated following self-harm or assault will have a positive BAC. Injury to the head and neck (S00–S19) was associated with a positive BAC, being related to assault.^15^ BAC should be requested when clinicians judge it would be helpful, but our study highlights the presentations that are more likely to be associated with an elevated BAC, for which we recommend a BAC be included in the initial tests requested. We also recommend that positive BAC results be followed up by brief advice/brief intervention when the patient is no longer in the RR.^1^ Although a positive BAC can help a clinician clarify the role that alcohol use may have played in the patient’s presentation, a positive BAC alone is not a sound basis for making judgements about the cause of the patient’s presenting complaint. A negative, or low, BAC is a definitive exclusion for reduced consciousness.

Patients presenting with trauma showed no relationship between severity, as defined by trauma scoring using the TRISS methodology^14^ and BAC level. Findings from different studies examining the relationship between alcohol and injury severity continue to be conflicting.^16^ Of the 196 trauma cases reported here, only 27 (14%) had a trauma score of 12 or more.

Requesting a BAC should be according to clinical judgement as for any investigation,^1^ but for the five presentations of collapse due to alcohol/drugs, self-harm, trauma, gastrointestinal bleeding and non-cardiac chest pain, we suggest BAC should be part of the initial set of blood tests obtained from the patients in the RR. We have demonstrated a higher rate of return visits and a trend towards higher mortality in the 6 months following a BAC-positive patient’s treatment in the RR. Davidson *et al*^17^ have previously confirmed poor outcomes for intoxicated ED patients (BAC >100 mg/100 ml) over a subsequent 5-year period, but these were not specifically from the RR.^17^

All ED systems are a reflection of healthcare practice and medical culture; BAC is a universal standard, laboratory facilities permitting.^18^ On its own, BAC is not as effective for detection as a questionnaire;^19^ however, it is a simple method of detection in the RR that can lead to subsequent brief advice/intervention depending on the ED environment, time, training and availability.^20^ This approach has now been recognised in national guidance to level 1 trauma centres in the USA, stating that all patients must be screened for alcohol misuse and provided brief advice/intervention.^21^ Internationally, ED may need economic incentives to encourage similar service provision to reduce re-visits.^22^ A recent survey of 191 ED in England revealed that almost half do not have systems in place to allow BAC testing to occur.^18^

Our study is confined to RR patients initially attending a single urban ED in a metropolitan area. The extent to which these data can be generalised to other ED is unclear. Although drinking patterns in the area served by our ED are not atypical of those in other densely populated areas in the UK,^23^ levels of alcohol use among patients attending other ED RR are not known.

In our study BAC results were unavailable to the medical staff; BAC were not previously routinely requested from our RR, nor was BAC testing available on site. The results of testing remained confidential, follow-up (ie, brief advice) not yet organised.^1^ Those involved in collecting outcome data on investigations and return visits to the ED were also unaware of results in order to minimise the likelihood of bias. We therefore did not study the influences that BAC results may have had on patient management.^10^

As alcohol is metabolised at approximately 15 mg/100 ml per hour,^24^ the delay between consuming alcohol and BAC being measured on arrival at the RR means that our data are likely to underestimate levels of misuse. Patients have diminished ability to recall past consumption as BAC increases, so that self-report is increasingly unreliable as consumption increases.^25^ Chronic heavy drinkers have developed both pharmacological and psychological tolerance, whereas the non-dependent binge drinker is more vulnerable.^2^

It is simple to add BAC to an initial set of RR blood investigations, a fluoride-oxalate bottle being used for both sugar and BAC estimations. BAC provides an important and inexpensive means of identifying the possible role, or not, of alcohol in a patient’s presenting complaint and furthermore, may act as “an alert” to counter possible clinical inertia of the treating doctor.^26^ The health consequences of alcohol misuse for those treated in the RR are potentially even greater than those treated elsewhere in the ED. Such patients, providing they survive, may therefore be even more likely to contemplate change,^27^ and be keen to avoid future risky behaviour and untoward incidents. BAC should routinely be considered for all RR patients with collapse from alcohol/drugs, trauma and intentional self-harm as well as for gastrointestinal bleeding and non-cardiac chest pain.
